# Understanding the reactivity of polycyclic aromatic hydrocarbons and related compounds

**DOI:** 10.1039/d0sc00222d

**Published:** 2020-04-01

**Authors:** Israel Fernández

**Affiliations:** Departamento de Química Orgánica I, Centro de Innovación en Química Avanzada (ORFEO-CINQA), Facultad de Ciencias Químicas, Universidad Complutense de Madrid 28040-Madrid Spain israel@quim.ucm.es

## Abstract

This perspective article summarizes recent applications of the combination of the activation strain model of reactivity and the energy decomposition analysis methods to the study of the reactivity of polycyclic aromatic hydrocarbons and related compounds such as cycloparaphenylenes, fullerenes and doped systems. To this end, we have selected representative examples to highlight the usefulness of this relatively novel computational approach to gain quantitative insight into the factors controlling the so far not fully understood reactivity of these species. Issues such as the influence of the size and curvature of the system on the reactivity are covered herein, which is crucial for the rational design of novel compounds with tuneable applications in different fields such as materials science or medicinal chemistry.

## Abstract 2

This perspective article summarizes recent applications of the combined activation strain model of reactivity and the energy decomposition analysis methods to the study of the reactivity of polycyclic aromatic hydrocarbons and related compounds.

## Graphical abstract

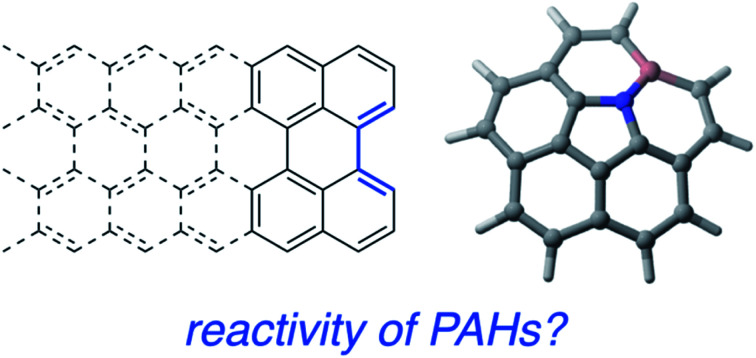

## 1. Introduction

Polycyclic aromatic hydrocarbons (PAHs) are a large family of organic compounds that are typically composed of two or more fused aromatic rings.^1,2^ These species, which are ubiquitous in modern life, can be divided into two main groups, namely planar PAHs, such as naphthalene, perylene or hexabenzocoronene, and curved PAHs, also known as bowl-shaped PAHs,^3–5^ such as corannulene or hemifullerene. The relevance and properties of these species are manifold. For instance, PAHs, particularly those having lower molecular weights, are important pollutants that exhibit a significant carcinogenic potency.^6,7^ On the other hand, many PAHs also possess interesting and tuneable optical and electrochemical properties, which are highly useful in materials science.^8^ Indeed, a good number of PAHs have been applied as semiconductor materials in organic field-effect transistors,^9^ light-emitting diodes,^10^ and even solar cells.^11^ In addition, PAHs are also ubiquitous components of organic matter in space accounting for a significant percentage of all carbon in the universe.^12^

From the above reasons, it becomes evident that understanding the intrinsic reactivity of PAHs is of crucial importance, especially for the rational design of novel PAHs with potential for application as organic materials. In this sense, different synthetic methods have been developed to produce new PAH derivatives,^13^ and among them cycloaddition reactions,^5,14^ transition metal catalysed arylations,^15^ aryne cyclotrimerizations,^16^ Scholl reactions,^17^ and flash vacuum pyrolysis^18^ should be particularly highlighted. Despite that, in many instances, the factors governing the reactivity of these species are poorly understood which severely hampers the development of new or existing methods for the preparation of novel derivatives with tuneable properties.

Over recent years, we have successfully applied the combined Activation Strain Model (ASM) of reactivity^19^ and Energy Decomposition Analysis (EDA)^20^ methods to provide a deeper and quantitative insight into those physical factors controlling the reactivity of PAHs and strongly related species such as cycloparaphenylenes or fullerenes. By means of representative recent applications, this perspective article summarizes the good performance of this relatively novel computational approach in exploring the chemistry of this important family of compounds.

## 2. The combined activation strain model and energy decomposition analysis approach

The Activation Strain Model (ASM) of reactivity has greatly contributed to our current understanding of fundamental transformations in chemistry, spanning from textbook processes in organic chemistry such as cycloaddition or S_N_2-reactions to transition metal-mediated reactions and biological processes.^19,21^ As this approach, also known as *distortion/interaction model*,^19*c*,22^ has been the focus of recent reviews,^19^ herein we briefly summarize the basics of this methodology.

The ASM is a systematic development of the Energy Decomposition Analysis (EDA)^20^ method (see below) proposed by Morokuma^23^ and Ziegler and Rauk^24^ to understand the nature of the chemical bonding in stable molecules. Within the ASM, the height of reaction barriers is described and understood in terms of the original reactants. Thus, the potential energy surface Δ*E*(*ζ*) is decomposed, along the reaction coordinate *ζ*, into the strain (Δ*E*_strain_(*ζ*)) that derives from the distortion of the individual reactants from their initial equilibrium geometries plus the actual interaction Δ*E*_int_(*ζ*) between the increasingly deformed reactants along the reaction coordinate (eqn (1)):

1 Δ*E*(*ζ*) = Δ*E*_strain_(*ζ*) + Δ*E*_int_(*ζ*)

It is the interplay between Δ*E*_strain_(*ζ*) and Δ*E*_int_(*ζ*) that determines if and at which point along *ζ* a barrier arises, namely, at the point where dΔ*E*_strain_(*ζ*)/d*ζ* = −dΔ*E*_int_(*ζ*)/d*ζ* is satisfied.

The ASM method can be combined with the EDA method to quantitatively partition the Δ*E*_int_(*ζ*) term.^19^ Within this approach, the total interaction between the reactants is further decomposed into the following chemically meaningful terms (eqn (2)):

2 Δ*E*_int_(*ζ*) = Δ*V*_elstat_(*ζ*) + Δ*E*_Pauli_(*ζ*) + Δ*E*_orb_(*ζ*) + Δ*E*_disp_(*ζ*)

where the term Δ*V*_elstat_ stands for the classical electrostatic interaction between the unperturbed charge distributions of the deformed reactants and is usually attractive. The Pauli repulsion Δ*E*_Pauli_ comprises the destabilizing interactions between occupied orbitals and is responsible for any steric repulsion. The orbital interaction Δ*E*_orb_ accounts for charge transfer (interaction between occupied orbitals on one moiety with unoccupied orbitals on the other, including HOMO–LUMO interactions) and polarization (empty–occupied orbital mixing on one fragment due to the presence of another fragment). Finally, the Δ*E*_disp_ term takes into account the interactions resulting from dispersion forces.

Moreover, the NOCV (Natural Orbital for Chemical Valence)^25^ extension of the EDA method can also be used for further partitioning the Δ*E*_orb_ term. The EDA-NOCV approach provides pairwise energy contributions of each pair of interacting orbitals to the total bond energy. Therefore, the EDA-NOCV scheme provides not only qualitative but also quantitative information about the strengths of the most significant orbital interactions between the interacting reactants along the reaction coordinate.

## 3. Reactivity of planar and curved polycyclic aromatic hydrocarbons

### (a) Reactivity of planar PAHs: towards the graphene limit

**Scheme 1:**
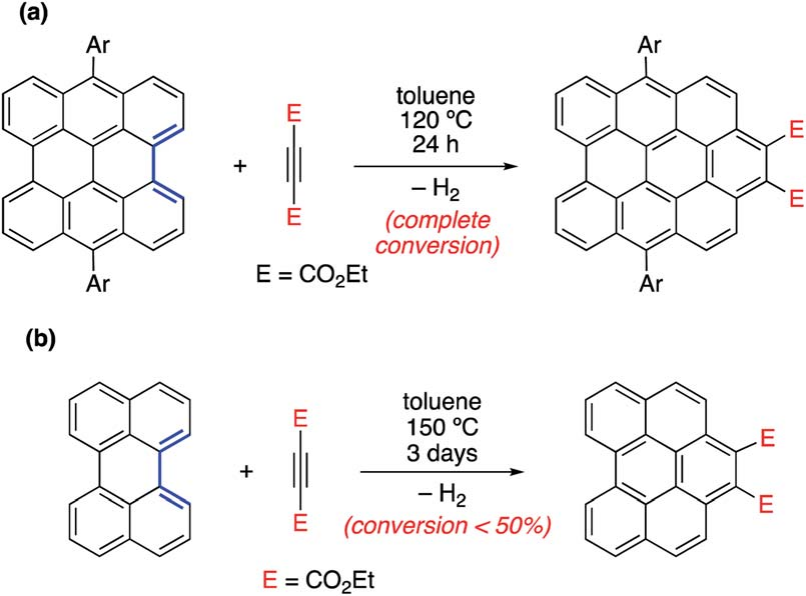
Diels–Alder cycloaddition reactions involving 7,14-dimesitylbisanthene (a) and perylene (b) described by Scott and co-workers (see ref. 26*a*).

In different studies,^26^ Scott and co-workers found that the Diels–Alder reactivity in the bay region of PAHs, a metal-free synthetic strategy proposed to grow carbon single-walled armchair nanotubes,^26*a*^ increases with an increase in the size of the system. Thus, whereas the cycloaddition reaction involving 7,14-dimesitylbisanthene and diethyl acetylenedicarboxylate proceeds with complete conversion at 120 °C for 24 h, a much lower conversion (<50%) was observed for perylene, even when the reaction was conducted at 150 °C for 72 h (Scheme 1).^25*a*^ Although this size-dependent reactivity has been traditionally ascribed to the nature of the conjugated double bonds in the bay region of the system (*i.e.* they more and more resemble 1,3-butadiene with an increase in the size of the PAH), very little was known about the factors controlling this clear reactivity trend until our study on the Diels–Alder reactivity of planar PAHs spanning from small systems such as biphenyl or phenanthrene to much larger species such as peripentacene or tetrabenzoovalene.^27^

**Fig. 1:**
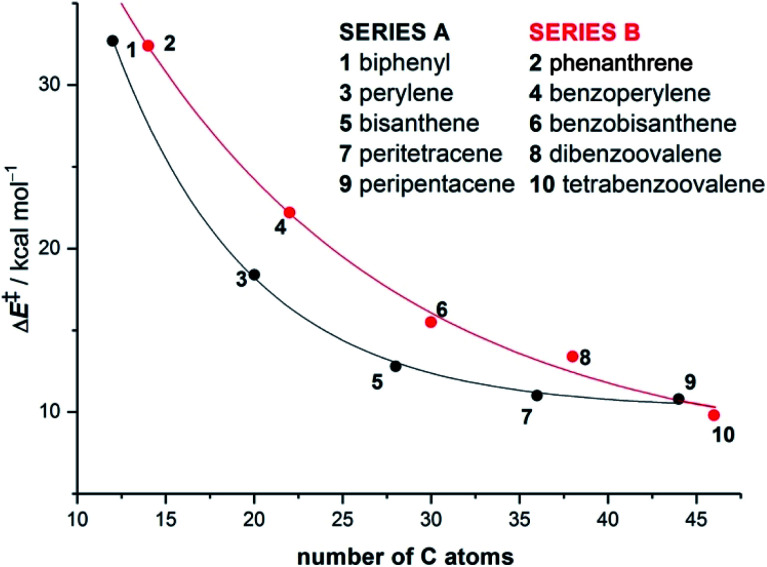
Plot of the activation barriers *versus* the total number of carbon atoms of planar PAHs **1–10** for the corresponding Diels–Alder reactions with maleic anhydride. Energies were computed at the BP86-D3/def2-TZVPP//RI-BP86-D3/def2-SVP level.

Our calculations indicate that, regardless of the size of the initial PAH, the Diels–Alder reaction with maleic anhydride occurs in a concerted manner through highly synchronous transition states (with the notable exception of the smallest systems whose corresponding transition states are much more asynchronous).^27^ The corresponding activation barriers steadily decrease when the size of the system is increased. In addition, the transformation becomes more and more exothermic, which is fully consistent with the experimentally observed reactivity enhancement. Interestingly, the change in both energies when going from one PAH to another follows an exponential decay converging toward a final value which seems to be reached for a system having 48–52 atoms (*i.e.* 18–20 fused six-membered rings) in its structure (Fig. 1). Therefore, we can predict, based on this asymptotic behaviour, a limit for the analogous cycloaddition reaction involving the bay region of a nanographene of Δ*E*^‡^ ≈ 10 kcal mol^−1^ and Δ*E*_R_ ≈ −30 kcal mol^−1^.

**Fig. 2:**
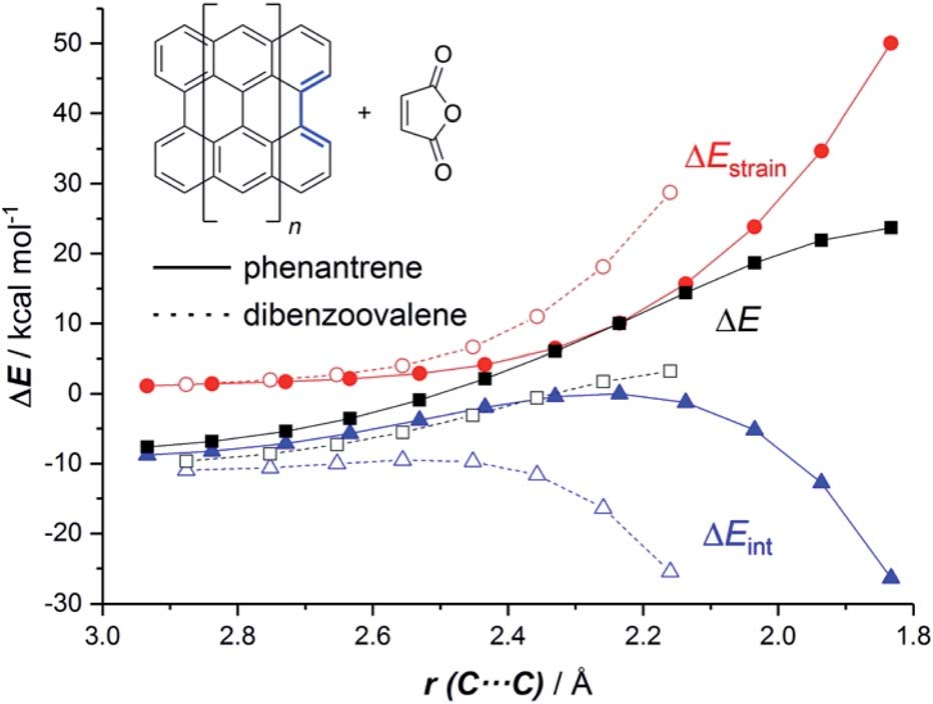
Comparative activation strain diagrams for the Diels–Alder cycloaddition reactions involving maleic anhydride and phenanthrene (solid lines) and dibenzoovalene (dashed lines) along the reaction coordinate projected onto the forming C⋯C bond. All data were computed at the BP86-D3/def2-TZVPP//RI-BP86-D3/def2-SVP level.

The ASM of reactivity was very helpful in understanding the above reactivity trend. Fig. 2 shows the Activation Strain Diagrams (ASDs) computed for the cycloaddition reactions involving phenanthrene and dibenzoovalene with maleic anhydride from the initial stages of the processes up to the corresponding transition states. As seen in Fig. 2, both systems exhibit rather similar ASDs in the sense that the interaction energy between the reactants (measured using Δ*E*_int_) becomes clearly stabilizing at the transition state region, a behaviour which is also found in related Diels–Alder cycloadditions and other pericyclic reactions.^28^ Despite that, it becomes evident that whereas the strain energy (Δ*E*_strain_) is less destabilizing for the smaller PAH (*i.e.* it requires less deformation energy to adopt the transition state geometry), the interaction energy is markedly stronger for the process involving dibenzoovalene along the entire reaction coordinate. Therefore, the interaction energy between the deformed reactants is solely the factor controlling the enhanced Diels–Alder reactivity of the larger PAHs.

**Fig. 3:**
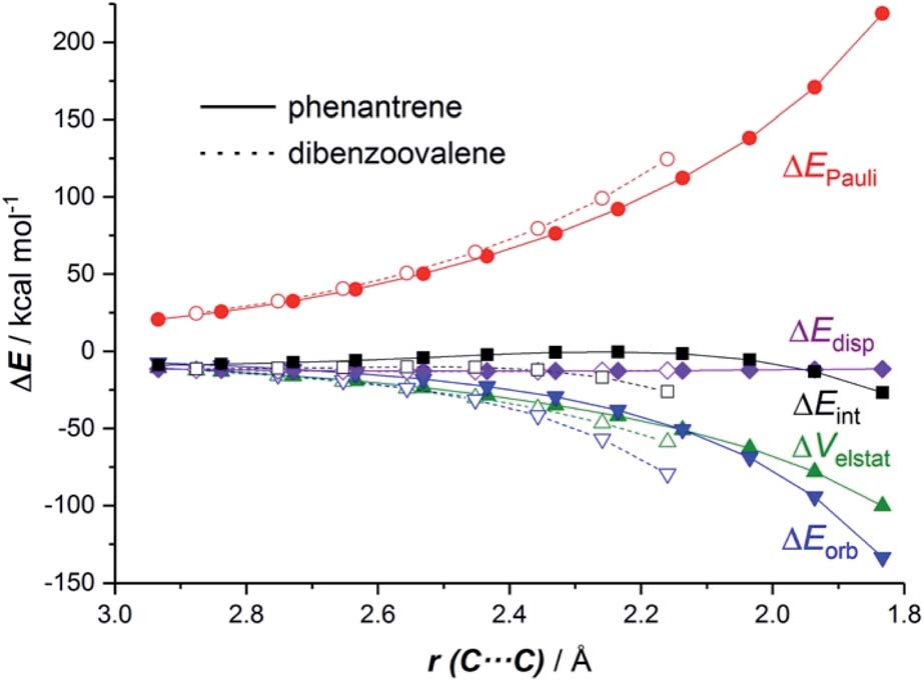
Comparative energy decomposition analyses for the Diels–Alder cycloaddition reactions involving maleic anhydride and phenanthrene (solid lines) and dibenzoovalene (dashed lines) along the reaction coordinate projected onto the forming C⋯C bond. All data were computed at the BP86-D3/TZ2P//RI-BP86-D3/def2-SVP level.

The EDA-NOCV method allowed us to understand the origin of the stronger Δ*E*_int_ computed for the cycloaddition involving the larger planar PAH dibenzoovalene. As graphically shown in Fig. 3, although the phenanthrene system benefits from a less destabilizing Pauli repulsion (Δ*E*_Pauli_), the attractive orbital (Δ*E*_orb_) and electrostatic (Δ*V*_elstat_, although to a much lesser extent) interactions are stronger (*i.e.* more stabilizing) for the reaction involving dibenzoovalene than for the analogous process involving its smaller counterpart.

**Fig. 4:**
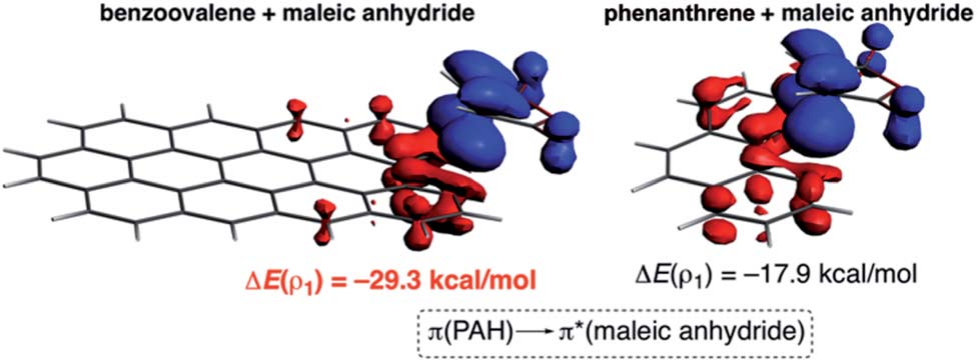
Plot of the deformation densities Δ*ρ* of the pairwise orbital interactions between maleic anhydride and dibenzoovalene (left) and phenanthrene (right) and the associated stabilization energies Δ*E*. The colour code of the charge flow is red → blue.

The NOCV extension of the EDA method indicates that two main molecular orbital interactions dominate the Δ*E*_orb_ term, namely the π(PAH) → π*(dienophile) interaction and the reverse π(dienophile) → π*(PAH) interaction. Not surprisingly, the former interaction is much stronger than the latter, which confirms the normal electron demanding nature of the considered Diels–Alder cycloadditions. Interestingly, both molecular orbital interactions, and especially the direct π(PAH) → π*(dienophile) interaction, are significantly stronger in the process involving dibenzoovalene (Fig. 4). As a result, the total Δ*E*_orb_ term is stronger for this reaction, which is translated into the computed stronger interaction between the reactants and ultimately, to a lower activation barrier. Therefore, the ASM-EDA(NOCV) method identifies the stronger orbital interactions in the cycloadditions involving larger planar PAHs as the main origin of the enhanced reactivity of these systems compared to their lighter counterparts.

### (b) Reactivity of bowl-shaped PAHs: relationship with C_60_

The Diels–Alder cycloaddition reaction has also been chosen as a representative reaction to understand the effect of the size and curvature on the reactivity of π-curved PAHs.

**Fig. 5:**
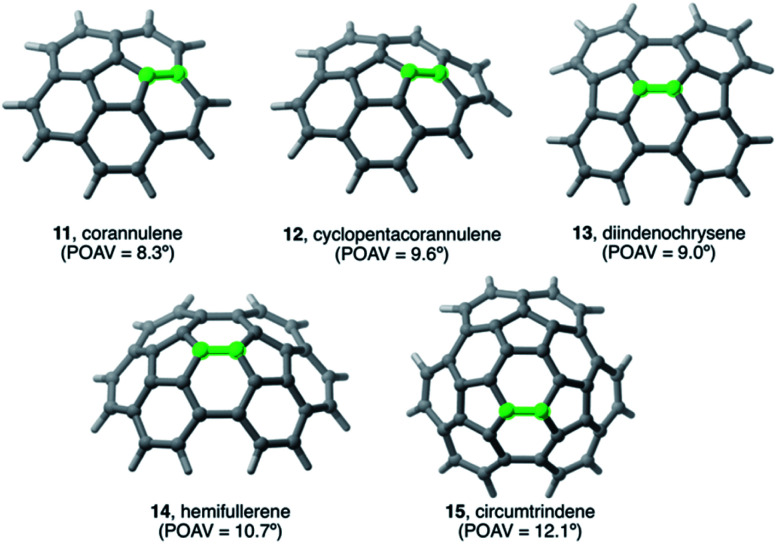
Curved PAHs considered in our study. The reactive [6,6]-bond is highlighted in green (see also ref. 32).

In general, it is found that, similar to planar PAHs, larger systems are systematically more reactive than their smaller counterparts.^5,29^ The reactivity of these curved systems has been traditionally rationalized by applying Fukui's Frontier Molecular Orbital (FMO) theory^30^ and the degree of pyramidalization of the trigonal carbon atoms, which can be quantitatively expressed in terms of the angle between the p_π_-orbital axis vectors, also known as the POAV index.^31^ However, these approaches are not always reliable reactivity descriptors for these species, as recently highlighted by Scott.^5,29^ For this reason, we decided to apply our ASM-EDA approach to gain further insight into the factors controlling the reactivity of these species.^32^ To this end, we investigated the Diels–Alder reactions between cyclopentadiene and the interior atoms of the bowl-shaped PAHs depicted in Fig. 5, which, similar to C_60_-fullerene,^33,34^ regioselectively produce the corresponding [6,6]-cycloadduct (*i.e.* the reactive C=C double-bond of the PAH is that which is shared by two adjacent six-membered rings).^5,32^

**Fig. 6:**
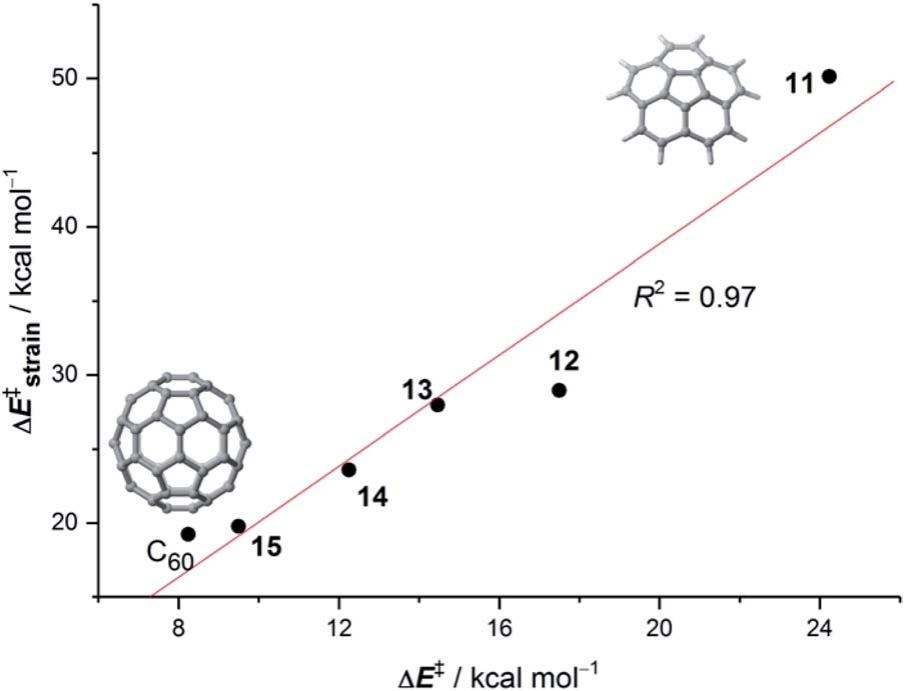
Plot of the computed activation barriers *versus* the activation strain energies for the Diels–Alder reaction involving bowl-shaped PAHs **11–15** and C_60_ and cyclopentadiene. All data were computed at the BP86-D3/def2-TZVPP//RI-BP86-D3/def2-SVP level.

Our calculations indicate that, starting from corannulene, there is a smooth convergence to the C_60_ energy barrier if the size of the buckybowl is increased. Therefore, both planar and curved PAHs exhibit a similar reactivity trend, *i.e.* the Diels–Alder reactivity is enhanced with an increase in the size of the system. Despite that, the ASM of reactivity suggests a different origin for this reactivity trend in the case of π-curved PAHs. As shown in Fig. 6, there is a clear linear relationship between the computed activation barriers and the corresponding activation strain energies, 
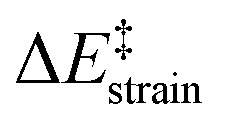
 (*i.e.* the energy required to deform the reactants from their equilibrium geometries to the geometry adopted at the transition state). This finding indicates that larger PAHs already possess a more curved equilibrium geometry which better fits into the geometry of the corresponding transition state. This required lower deformation energy is then translated into the lower activation barriers computed for the cycloaddition reactions involving larger PAHs than those for their smaller counterparts. A similar conclusion, *i.e.* strain is the key factor controlling the reactivity of curved PAHs, was found by Osuna and Houk^35^ in a related exhaustive study on the Diels–Alder cycloaddition reactions of *s-cis*-1,3-butadiene to the different bonds of, among others, corannulene, coronene, and two derivatives that involved four additional five-membered rings added to the periphery of both PAHs to increase their curvature. In that study, the authors also found that the activation strain energy nicely correlates with the barrier heights of the corresponding cycloaddition reactions, therefore confirming the important role of the initial curvature of the PAH in its reactivity.

## 4. Reactivity of related species: pyrenophanes, cycloparaphenylenes and larger systems

The results above confirm that the size and curvature have a strong influence on the reactivity of PAHs. To further explore the impact of the initial geometry of the system on the barrier heights, we explored the reactivity of related curved systems such as pyrenophanes and cycloparaphenylenes.

### (a) Reactivity of pyrenophanes

**Scheme 2:**
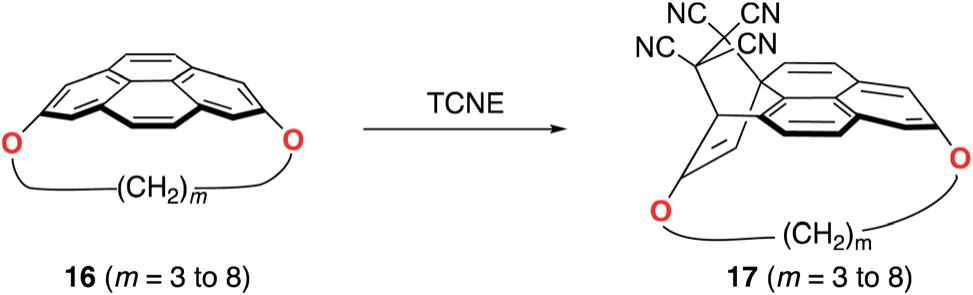
Diels–Alder reactions involving (2,7)-pyrenophanes **16** and TCNE.

Pyrenophanes are a subgroup of cyclophanes where two nonadjacent positions of pyrene are bridged by an aliphatic chain.^36^ These species have attracted much attention recently mainly because of the remarkable photophysical and photochemical properties associated with the pyrene nucleus.^37,38^ In particular, Bodwell and co-workers prepared a series of [*n*](2,7)pyrenophanes and studied their reactivity in order to prepare new π-curved organic materials.^36,39^ It was found that the tether connecting the 2 and 7 positions of pyrene has a strong influence on the Diels–Alder reactivity of the system in the sense that systems having long tethers (*n* = 7, 8) are typically less reactive than those having shorter bridges. Although it is suggested that this reactivity trend is mainly caused by the strain relief during the transformation,^39^ the ultimate factors controlling the reactivity of these interesting species are not completely understood. For this reason, we explored the influence of the length of the tether on the Diels–Alder reactivity of the (2,7)pyrenophanes depicted in Scheme 2 with tetracyanoethylene (TCNE) as the dienophile (the species used by Bodwell and co-workers in the experiments).^40^

**Fig. 7:**
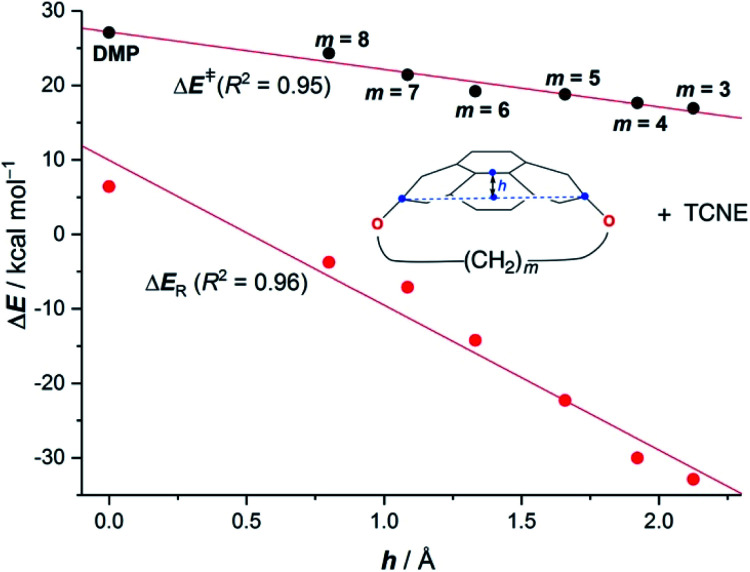
Plot of the computed activation barriers (Δ*E*^‡^) and reaction energies (Δ*E*_R_) for the Diels–Alder reaction involving TCNE and pyrenophanes **16***versus* the curvature parameter *h*. Inset: geometrical definition of parameter *h* (defined as the distance between the center of the bond connecting the central C3a^1^ and C5a^1^ atoms and the center of the line connecting C2 and C7 atoms of the pyrane moiety). Energy values were computed at the PCM(benzene)-M06-2X/def2-TZVPP//B3LYP-D3/def2-SVP level.

Our calculations clearly indicate that regardless of the length of the bridge, the cycloadditions involving pyrenophanes proceed systematically with a lower barrier and are much more exothermic than the analogous reaction involving the planar 2,7-dimethoxypyrene (DMP) counterpart. This finding strongly suggests that the reactivity of these species is strongly dominated by the curvature of the system. Indeed, very good linear relationships were found when plotting either the activation barriers or reaction energies *versus* the curvature of the initial pyrenophane, which can be measured using the geometrical parameter *h* (see Fig. 7 for a definition). This indicates that pyrenophanes with longer tethers possess a low *h* value, which is then translated into a higher activation barrier. The opposite is found for the systems having shorter tethers (with higher curvatures), which is fully consistent with the experimental observations (see above).^36,39^

**Fig. 8:**
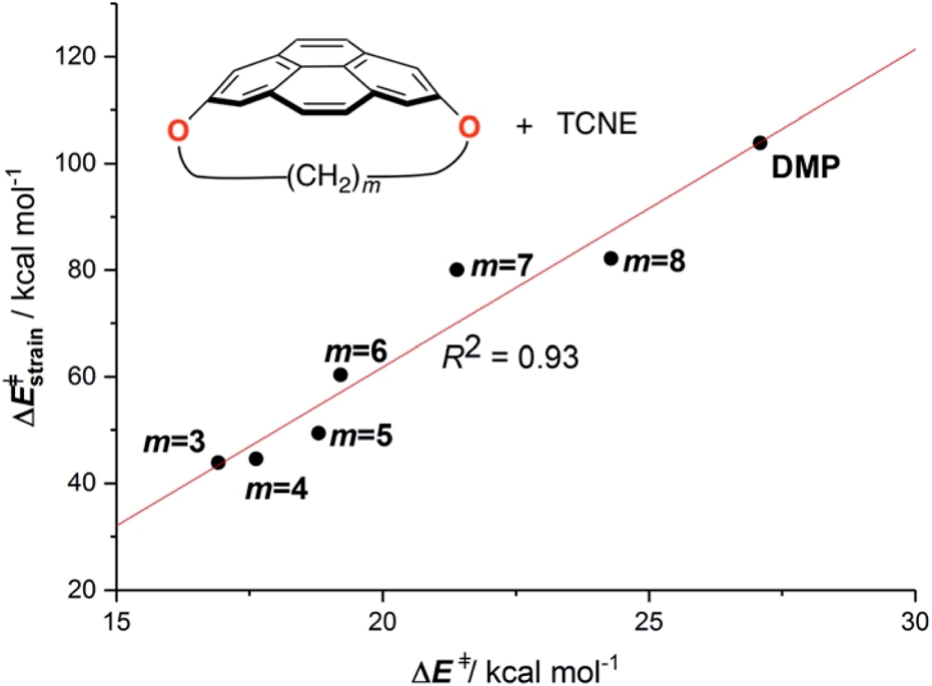
Plot of the computed activation barriers *versus* the activation strain energies for the Diels–Alder reaction involving pyrenophanes **16** and TCNE. All data were computed at the PCM(benzene)-M06-2X/def2-TZVPP//B3LYP-D3/def2-SVP level.

The above plot suggests that the initial bent equilibrium geometry plays a crucial role in determining the reactivity of the (2,7)pyrenophanes. Similar to the bowl-shaped PAHs described above, it is expected that the origin of the lower barriers computed for the processes involving the more bent systems can be found in a much lower strain energy to adopt the corresponding transition state structures. To our delight, a nice linear relationship was found when plotting the computed activation barriers and the corresponding activation strain energies, 
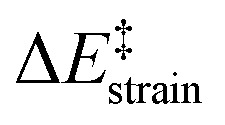
 (Fig. 8). This confirms that the systems having higher curvature values already possess a bent initial geometry which better fits into the corresponding transition state geometry, and therefore require a lower deformation energy. In this sense, it is not surprising that the transition states associated with these systems are reached systematically earlier than those associated with pyrenophanes having low *h* values (*i.e.* less bent and with longer bridges).

### (b) Reactivity of strained-alkyne embedded cycloparaphenylenes

**Scheme 3:**
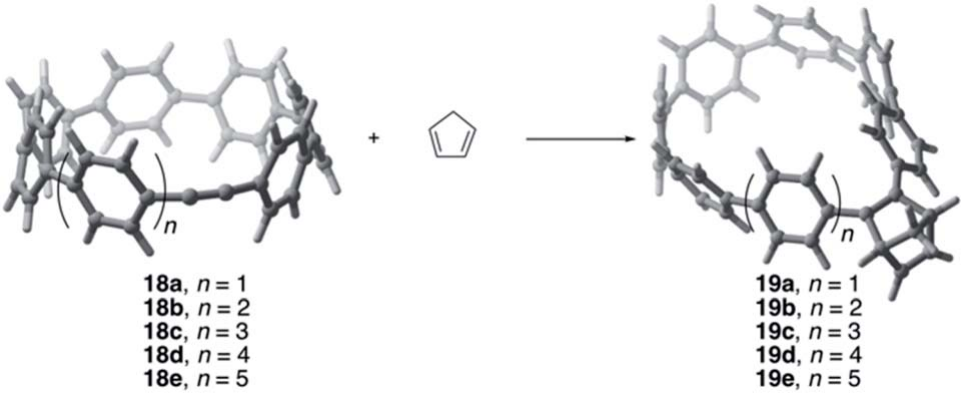
Diels–Alder reactions involving alkyne-embedded cycloparaphenylenes and cyclopentadiene.

Very recently, Jasti and co-workers prepared a series of strained-alkyne cycloparaphenylenes (CPPs)^41^ whose size and reactivity can be precisely tuned.^42,43^ Similar to the pyrenophanes described above, it was experimentally found that the reactivity of the system having seven phenylenes in its structure (**18a**) is markedly higher than that of its larger counterparts (**18c** and **18e**, Scheme 3). We then decided to apply our ASM-EDA approach to shed more light on the factors controlling the reactivity of these species,^44^ which is crucial for the design of new strained “clickable” and radially oriented π-rich macrocycles.^43^ To this end, we considered the Diels–Alder cycloaddition reaction involving cyclopentadiene and the alkyne embedded CPPs **18a–e**, having seven to eleven phenylenes in their structures.

Similar to the reactivity of pyrenophanes, our calculations confirm that the reactivity of these CPPs steadily decreases with an increase of the size of the system up to the limit of the corresponding planar counterpart diphenylacetylene (DPA). This indicates, once again, that the curvature of the system governs the reactivity of these species in the sense that more bent systems (*i.e.* smaller systems) are systematically more reactive than their larger congeners. Indeed, very good linear relationships were found again when plotting either the computed activation barriers or reaction energies *versus* the curvature *θ*, defined as the difference between the C–C≡C angle in the linear DPA (180°) and the corresponding angle in the macrocycles **18** (correlation coefficient *R*^2^ = 0.99 and 0.92, respectively).^44^

**Fig. 9:**
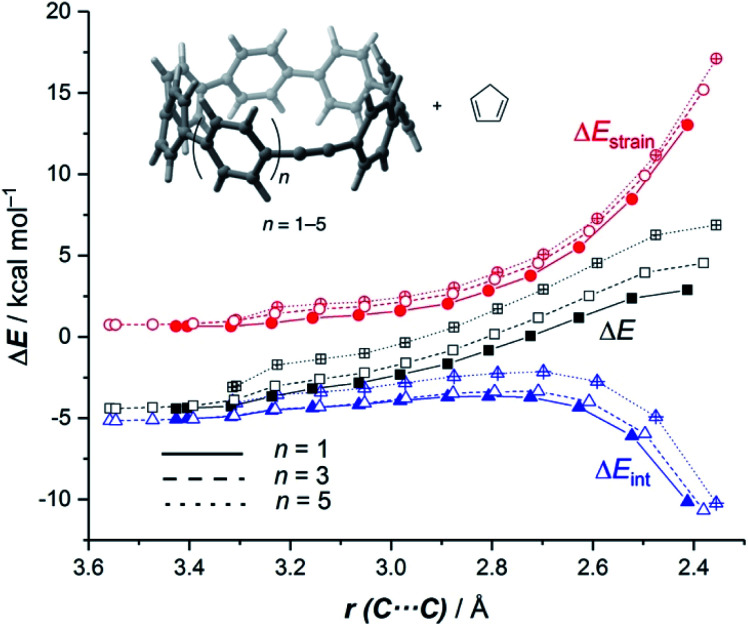
Comparative activation strain diagrams for the Diels–Alder cycloaddition reactions involving cyclopentadiene and CPPs **18a** (*n* = 1, solid lines), **18c** (*n* = 3, dashed lines) and **18e** (*n* = 5, dotted lines) along the reaction coordinate projected onto the forming C⋯C bond. All data were computed at the BP86-D3/def2-TZVPP//RI-BP86-D3/def2-SVP level.

It is again expected that the most curved systems require lower deformation energies, and as a consequence, they exhibit an enhanced reactivity compared to their less curved congeners. According to the ASDs for the processes involving **18a**, **18c** and **18e**, this hypothesis is confirmed as the Δ*E*_strain_ curve is clearly less destabilizing for **18a** than for **18c** and **18e** along the entire reaction coordinate (Fig. 9). Despite that, the ASDs suggest that, at variance with other curved PAHs, the difference in reactivity of these CPPs is mainly dominated by the interaction term rather than the deformation energy. For instance, at the same consistent C⋯ C bond forming distance of 2.5 Å, the difference in the Δ*E*_int_ term (ΔΔ*E*_int_ = 1.2 and 2.6 kcal mol^−1^ for **18c** and **18e**, with respect to **18a**, respectively) roughly matches the difference in the total energy (ΔΔ*E* = 1.6 and 3.3 kcal mol^−1^). Therefore, it can be concluded that the initial bent geometry of the system not only leads to a reduced strain energy but also enhances the interaction energy between the deformed reactants.^45^ This is, according to the EDA method, mainly ascribed to the combination of both stronger electrostatic and orbital interactions.

The ASM-EDA(NOCV) approach has also been particularly useful to understand the reactivity of related larger systems such as fullerenes and carbon nanotubes. In the chemistry of fullerenes, issues such as the influence of the encapsulation of ions or molecules inside the fullerene cage on both the reactivity and regioselectivity have been studied by our group.^46,47^ Regarding nanotubes, Houk, Lan, and co-workers reported that the interaction energy becomes the major factor controlling the reactivity of single-walled carbon nanotubes of different diameters (4–9,5).^48^ In a related study, Solà and co-workers explored the influence of the curvature of single-walled carbon nanotubes on their Diels–Alder reactivity with benzyne.^49^ In this case, it was found that the deformation of the initial reactants in the rate-determining transition states is the key factor governing the chemoselectivity of the process.

## 5. Influence of the presence of heteroatoms on the reactivity: doped systems

The integration of heteroatoms, especially those belonging to groups 13–16, into the framework of PAHs constitutes a really useful way to modulate their properties.^50^ Indeed, new systems having potential for application in the fabrication of biomedical and optoelectronic materials have been produced as a result of replacing carbon atoms with heteroatoms. Not surprisingly, such a replacement induces a significant modification of the electronic structure of the system which, of course, greatly affects its reactivity. Despite that, very little is known about the actual influence of the presence of heteroatoms in the structure of PAHs and related systems on their reactivity.

In this sense, we recently investigated the reactivity of parent 1,2-borazines and related group 15 and 16 analogues, where a C=C group in benzene was replaced by an isoelectronic BN fragment.^51^ Such C=C/BN replacement in aromatic molecules is particularly attracting considerable interest in materials chemistry and medicinal chemistry.^52^ For instance, Liu and co-workers reported that, different to benzene, the analogous *N*-TBS-B-Me-1,2-azaborine (TBS = *tert*-butyldimethylsilyl) is able to undergo irreversible Diels–Alder reactions with electron-deficient dienophiles such as maleic anhydride or *N*-methylmaleimide in the presence of AlCl_3_ as a catalyst at room temperature and with complete *endo*-diastereoselectivity.^53^ Our ASM calculations indicate that the enhanced Diels–Alder reactivity of the 1,2-azaborine systems compared to benzene finds its origin not only in a lower strain energy but mainly in the much stronger interaction energy between the reactants along the entire reaction coordinate.^51*a*^ This can be ascribed to the reduced aromaticity strength in the system induced by the presence of the BN moiety, which makes the 1,2-azaborine a much better diene than the much more aromatic benzene.^51*b*^

**Fig. 10:**
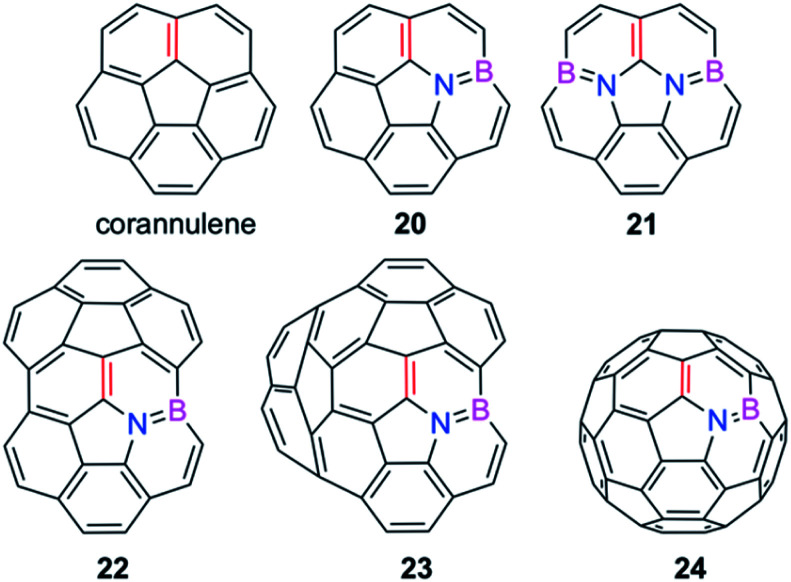
BN-embedded curved PAHs considered in our study. The most reactive [6,6]-bond is highlighted in red (see also ref. 54).

We extended these findings in a recent study aimed to understand the impact of the C=C/B–N replacement on the reactivity of π-curved PAHs.^54^ Compared to their BN-embedded planar congeners, these doped curved systems have been comparatively much less explored very likely due to the experimental difficulties associated with their preparation and the lack of knowledge of their intrinsic reactivity. For this reason, we first compared the Diels–Alder reactivity, using cyclopentadiene as the diene, of the parent corannulene with its BN-analogues **20** and **21** (the latter being a model of the experimentally prepared 10b^1^,18b^1^-diaza-10b,18b-diboratetrabenzo[*a*,*g*,*j*,*m*]corannulene)^55^ (see Fig. 10).

**Fig. 11:**
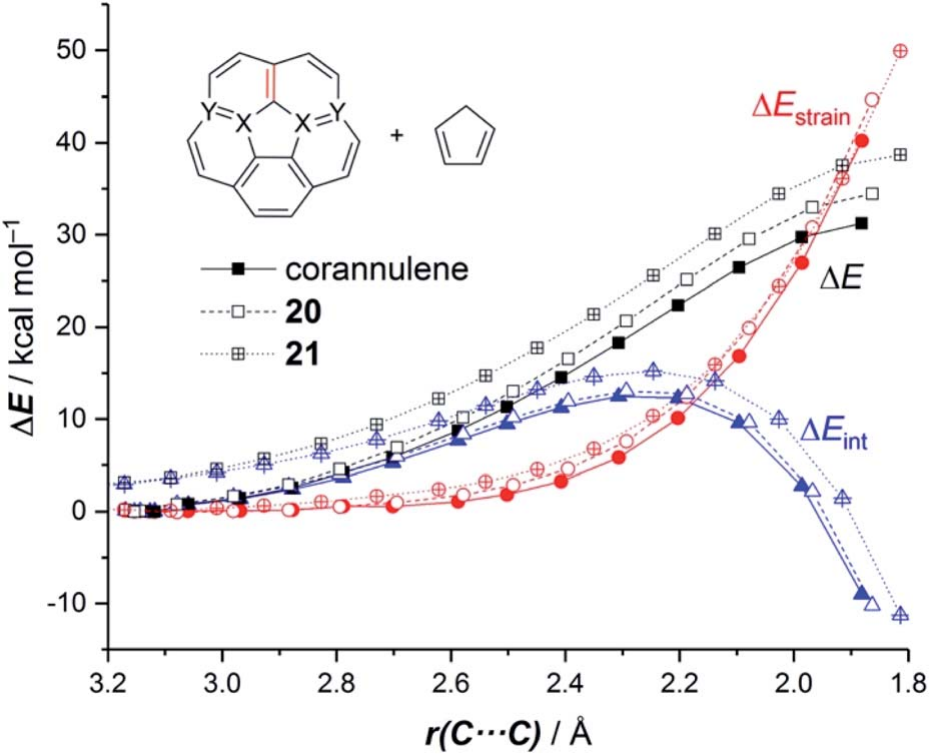
Comparative activation strain diagrams for the Diels–Alder cycloaddition reactions involving cyclopentadiene and corannulene (solid lines), BN-corannulene **20** (dashed lines) and (BN)_2_-corannulene **21** (dotted lines) along the reaction coordinate projected onto the forming C⋯C bond. All data were computed at the M06-2X/def2-TZVPP//M06-2X/def2-SVP level.

It was found that the presence of the BN fragment induces a significant planarization of the system (bowl-depth decreases from 0.88 Å in corannulene to 0.68 Å in **20**, and to 0.26 Å in **21**) which is translated into a remarkable reduction of the corresponding bowl-to-bowl inversion barrier. This structural modification results also in a markedly reduced Diels–Alder reactivity with cyclopentadiene (reactivity order: corannulene > **20** > **21**). According to the ASM approach, the more curved corannulene benefits from both a less destabilizing strain energy and a stronger interaction between the deformed reactants, which is translated into the computed higher reactivity of this system (Fig. 11). This finding is directly connected to that observed in the reactivity of other curved systems such as strained-alkyne embedded cycloparaphenylenes (see above), where the initial curvature (*i.e.* pre-distortion) of the system is not only translated into a lower deformation energy but also into a stronger interaction between the reactants. The application of the EDA(NOCV) method indicates that the enhanced interaction computed for the process involving corannulene is caused by both stronger electrostatic and orbital (mainly HOMO_diene_–LUMO_corannulene_) interactions in a nearly identical manner.^54^

**Fig. 12:**
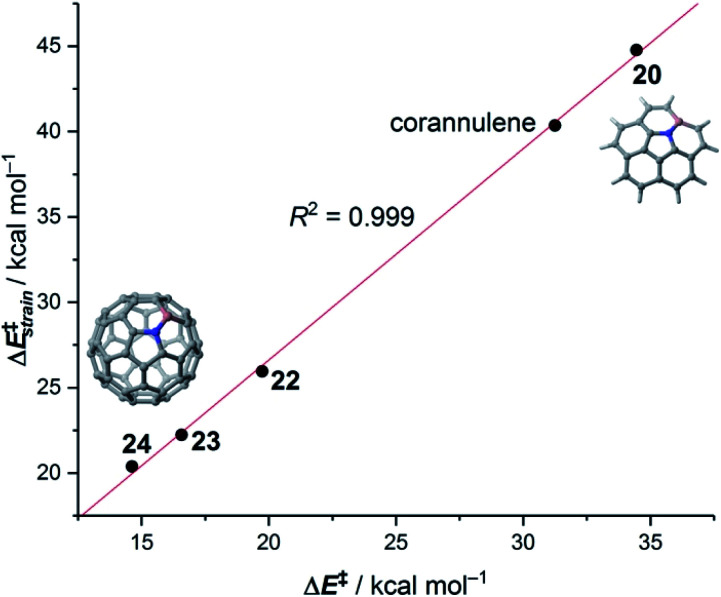
Plot of the computed activation barriers *versus* the activation strain energies for the Diels–Alder reaction involving BN-embedded curved PAHs **20–24** and cyclopentadiene. All data were computed at the M06-2X/def2-TZVPP//M06-2X/def2-SVP level.

The crucial role of the initial curvature of the system was further confirmed when considering the reactivity of related BN-embedded larger curved PAHs such as BN-hemifullerene **22**, BN-circumtrindene **23** and even BN-fullerene **24** (see Fig. 10). Not surprisingly, a steady increase of the Diels–Alder reactivity was found as a consequence of the increasing curvature of the system when going from corannulene or BN-corannulene (less curved systems) to BN-circumtrindene or BN-fullerene. Indeed, a perfect linear correlation, similar to that found for their all-carbon analogues (see Fig. 6), was found when plotting the computed activation barriers *versus* the corresponding activation strain energies, 
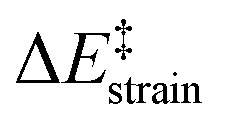
 (correlation coefficient *R*^2^ = 0.999, Fig. 12). This confirms, once again, that curved PAHs require less deformation energy to adopt the corresponding transition state geometry, which results in lower barrier processes than for the reactions involving less curved systems.

**Fig. 13:**
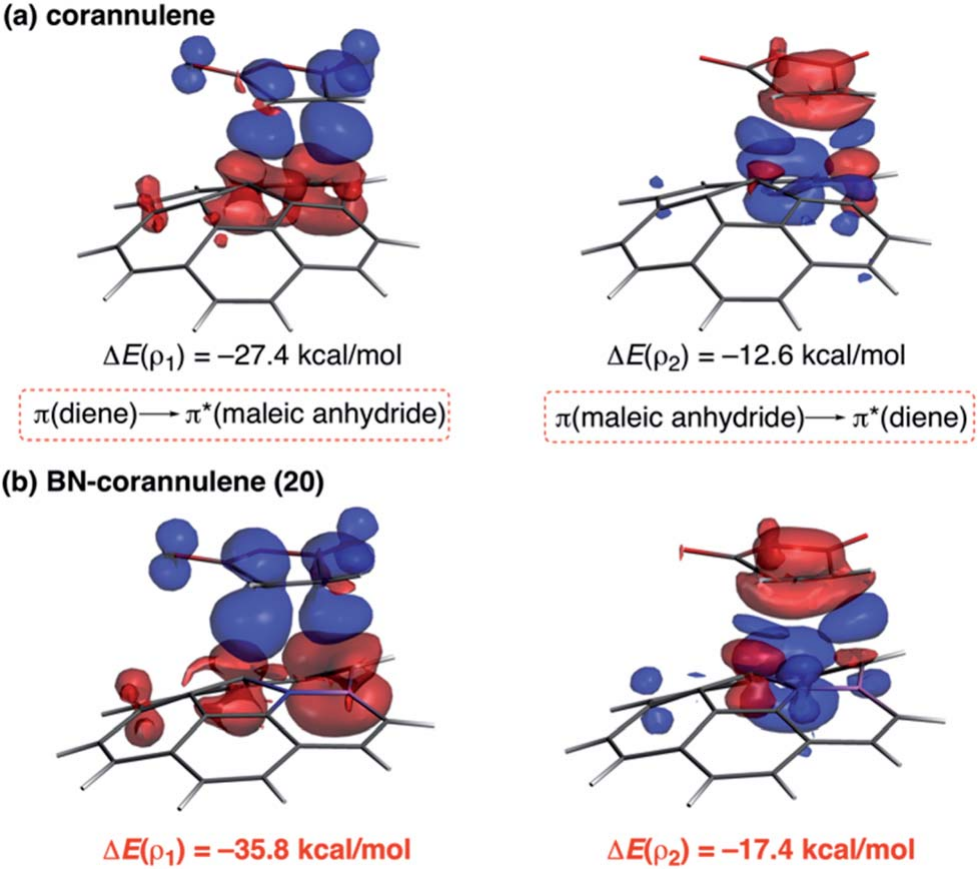
Plot of the deformation densities Δ*ρ* of the pairwise orbital interactions between maleic anhydride and corannulene (a) and BN-corannulene **20** (b) and associated stabilization energies Δ*E*. The color code of the charge flow is red → blue.

As commented above, the replacement of the C=C moiety by BN in benzene makes the system a better diene. A similar effect is also found in these π-curved systems. Indeed, at variance with the cycloaddition involving cyclopentadiene, BN-corannulene **20** reacts better (*i.e.* with a lower activation barrier and more exothermic reaction energy) than corannulene with a dienophile such as maleic anhydride. The ASM method indicates that the reactivity reversal of the BN-system is solely ascribed to the stronger interaction between the reactants along the entire reaction coordinate, which, according to the EDA method, is almost exclusively due to more stabilizing orbital interactions.^54^ The NOCV extension of the EDA method confirms that the stronger orbital interactions computed for the process involving **20** than for the parent corannulene derive from both the direct π(diene) → π*(dienophile) and the reverse π(dienophile) → π*(diene) molecular orbital interactions, which are comparatively weaker for the process involving corannulene (see Fig. 13). Therefore, it is confirmed that the replacement of a C=C fragment by an isoelectronic B–N moiety dramatically modifies the reactivity of the doped PAHs. Thus, whereas the all-carbon system tends to react as a dienophile in Diels–Alder cycloaddition reactions, its BN-counterpart is a better diene in the analogous process with maleic anhydride.^56^

The effect of the replacement of carbon atoms by heteroatoms was also studied in larger systems such as fullerenes. In particular, we explored the reactivity of azafullerenes, the only class of heterofullerenes that have been synthesized in macroscopic quantities so far^57^ and that, due to their exceptional energy- and charge-transfer properties, have been employed in organic solar cells.^57*c*,58^ Our calculations indicate that, compared to C_60_, the Diels–Alder reaction with cyclopentadiene involving its doped-counterpart C_59_NH azafullerene is both kinetically and thermodynamically less favoured.^59^ This decreased reactivity is ascribed by the ASM-EDA(NOCV) method exclusively to a remarkable reduction of the interaction between the deformed reactants. The presence of the nitrogen atom and the CH fragment significantly modifies the electronic structure of the fullerenic cage and weakens the direct π(cyclopentadiene) → π*(fullerene) molecular orbital interaction. This results in a lower total interaction and therefore, in a higher barrier cycloaddition than for C_60_.^59^ This study was also extended to charged systems, C_59_N^+^ and C_59_N^−^, species prepared or detected experimentally.^60,61^ Based on the computed barriers, the following Diels–Alder reactivity trend was found: C_59_N^+^ > C_60_ > C_59_NH > C_59_N^−^.^62^ Once again, the interaction energy between the reactants was found to be the key factor governing the reactivity of these azafullerenes. Despite that, the weaker Δ*E*_int_ computed for C_59_NH or C_59_N^−^ does not derive from weaker orbital interactions but from a more destabilizing Pauli repulsion between closed-shells as a consequence of the presence of two additional π-electrons in these systems compared to C_59_N^+^ or C_60_.^62^

**Fig. 14:**
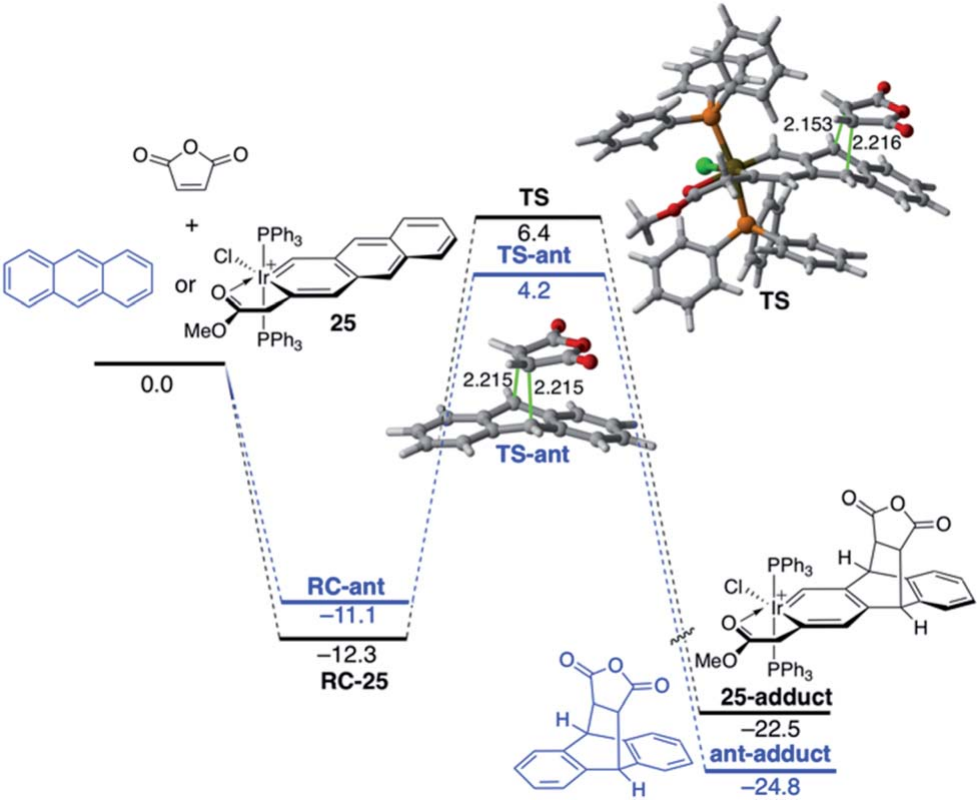
Computed reaction profile for the Diels–Alder cycloaddition reaction between maleic anhydride and anthracene (blue lines) and iridaanthracene **25** (black lines). Relative energies and bond distances are given in kcal mol^−1^ and angstroms, respectively. All data have been computed at the BP86-D3/def2-TZVPP//RI-BP86-D3/def2-SVP level.

The modification of the electronic structure and reactivity of PAHs is not restricted to the incorporation of group 13–16 heteroatoms. In fact, it can also be achieved by incorporating transition metal fragments in their structures instead. For instance, it was found that the central ring of metallaanthracenes, a particular group of metallabenzenes^63^ where a CH unit in anthracene is replaced by an isolobal transition-metal fragment, is systematically less reactive than the analogous ring of the parent anthracene in their Diels–Alder cycloaddition reactions with maleic anhydride.^64^ For instance, Fig. 14 shows the computed reaction profiles for the Diels–Alder reactions involving maleic anhydride and anthracene and iridaanthracene **25**, a species recently prepared by Frogley and Wright.^65^ As clearly seen, the cycloaddition involving **25** is both kinetically (ΔΔ*E*^‡^ = 3.4 kcal mol^−1^) and thermodynamically (ΔΔ*E*_R_ = 2.3 kcal mol^−1^) less favoured than the analogous process involving the parent anthracene.

**Fig. 15:**
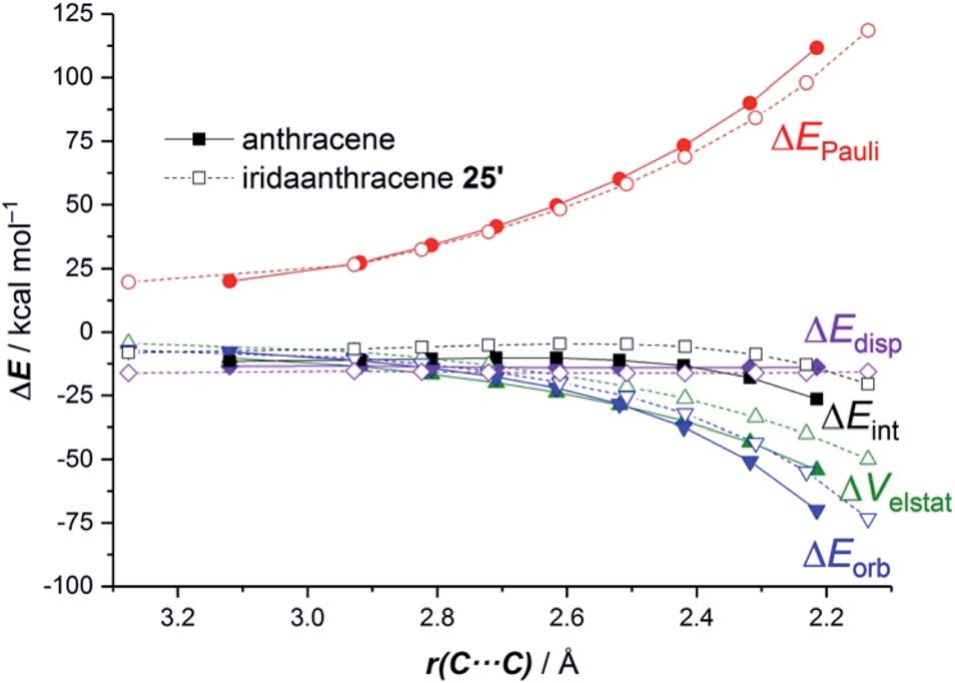
Comparative energy decomposition analyses for the Diels–Alder cycloaddition reactions involving maleic anhydride and anthracene (solid lines) and iridaanthracene **25′** (dashed lines, the bulky PPh_3_ ligands in **25** were replaced by PMe_3_ ligands) along the reaction coordinate projected onto the forming C⋯C bond. All data were computed at the ZORA-BP86-D3/TZ2P//RI-BP86-D3/def2-SVP level.

The ASM approach indicates that the cycloaddition involving anthracene, although requires a higher deformation energy, benefits from a much stronger interaction between the reactants along the entire reaction coordinate compared to the process involving its organometallic counterpart **25**.^64^ As graphically shown in Fig. 15, the EDA method indicates that this stronger interaction derives from more stabilizing electrostatic and orbital interactions, the latter resulting mainly from a stronger π(anthracene) → π*(maleic anhydride) molecular orbital interaction.

## 6. Conclusions and outlook

By means of selected representative applications, in this perspective article we have illustrated the good performance of the combined Activation Strain Model (ASM) of reactivity and Energy Decomposition Analysis (EDA) methods to provide a detailed rationalization of the physical factors controlling the reactivity of PAHs and strongly related species. Issues such as the influence of the size, curvature and the presence of heteroatoms in the system on their reactivity can be easily understood in a quantitative manner using this computational approach, which not only complements but, in many cases, also outperforms other more traditional methods based on the application of FMO arguments or POAV angles. In our opinion, the ASM-EDA(NOCV) methodology can be an extremely useful tool to guide experimentalists towards the development of methods for the preparation of novel PAH derivatives with tuneable properties and potential for application in materials science or medicinal chemistry.

## Conflicts of interest

There are no conflicts to declare.
